# Fetal Lung Cells Transfer Improves Emphysematous Change in a Mouse Model of Neutrophil Elastase-Induced Lung Emphysema

**DOI:** 10.3390/cimb44090269

**Published:** 2022-08-29

**Authors:** Shin Ohta, Akihiko Tanaka, Tomoko Okazaki, Hatsuko Mikuni, Tomoki Uno, Yoshitaka Uchida, Tomoyuki Kimura, Yosuke Fukuda, Megumi Jinno, Kuniaki Hirai, Yoshito Miyata, Hideki Inoue, Tetsuya Homma, Mayumi Yamamoto, Shintaro Suzuki, Hironori Sagara

**Affiliations:** Department of Medicine, Division of Respiratory Medicine and Allergology, Showa University, Tokyo 142-8666, Japan

**Keywords:** lung emphysema, mouse model, neutrophil elastase, fetal lung cell, regeneration

## Abstract

Recently, several studies for lung regeneration have been reported. However, regenerating the lung tissue by the transfer of any cells directly to the lung has been hardly successful. The aim of this study was to evaluate the effect of fetal lung cells (FLCs) in a mouse model of lung emphysema. C57BL/6 mice were stimulated with neutrophil elastase (NE) intra-tracheally (i.t.) to generate lung emphysema. To collect fetal lung cells, C57BL/6-Tg (CAG-EGFP) mice were bred for 14 days. Before delivery, the bred mice were euthanized, and fetal lungs were harvested from the fetal mice and the cells were collected. The FLCs were transferred i.t. 24 h after the NE instillation. Four weeks after the NE instillation, mice were euthanized, and the samples were collected. The mean linear intercept (MLI) was significantly prolonged in the NE instillation group compared to the control group. However, in the FLCs transfer group stimulated with NE, the MLI became shorter than the NE-stimulated group without an FLCs transfer. This result shows that an FLCs transfer inhibited the progression of lung emphysema. Additionally, motility of the mice was also improved by the FLCs transfer. These results indicate that transfer of the FLCs, which were presumed to be progenitor cells for lung tissue, may improve the emphysematous change.

## 1. Introduction

In patients with chronic obstructive pulmonary disease (COPD), the destruction of lung tissue leads to emphysematous changes, resulting in impaired gas exchange, which causes chronic respiratory failure. As a result, dyspnea appears and their activity declines. Currently, there is no treatment to improve emphysematous changes once they occur, and hypoxia is treated only by the administration of oxygen.

Features of emphysema include the destruction of the alveolar walls, the loss of elastic recoils, and the abnormal expansion of the airspace from the peripheral to the terminal bronchioles [1]. The pathogenesis of emphysema involves alveolar macrophages, CD8+ T cells, and neutrophil-mediated inflammation, most commonly activated by cigarette smoke [2]. These cells produce several variants of protease; among them, neutrophil elastase (NE) has been reported as particularly important in the development of emphysema [3,4]. In fact, a previous study has shown that intra-tracheal (i.t.) administration of NE creates a mouse model of emphysema that exhibits emphysematous changes in the lungs [4].

Although there are several studies that have focused on regenerative treatments of the lung, the majority of such studies involving the transfer of cells directly to the lung have not shown benefit. Nevertheless, two studies have reported that the intravenous transfer of bone marrow cells or the i.t transfer of bone marrow-derived mesenchymal stem cells improved lipopolysaccharide-induced lung injury in a mouse model [5,6]. However, the suspected mechanism might have involved the induction of proliferation and differentiation of the host lung cells rather than the transferred cells themselves. Additionally, there are other reports showing that the intravenous transfer of embryonic lung cells may repair lung emphysema [7,8].

In the present study, we investigated whether the transfer of fetal lung cells (FLCs) directly into the lung in a mouse model of NE-induced emphysematous change (emphysema model mouse) could trigger lung tissue regeneration or an improvement in the emphysematous changes in the lung. The prenatal fetal lung is immature and extracted FLCs are thought to be progenitor cells undergoing differentiation into lung tissue. Therefore, we examined whether FLCs transferred i.t. into the emphysema model mouse could reverse the emphysematous changes and regenerate lung tissue.

## 2. Materials and Methods

### 2.1. Animal

Male C57BL/6 mice were obtained from the Saitama Experimental Animal Supply Co., Ltd. (Saitama, Japan). All animal experiments were approved by the Animal Care and Use Committee of the Showa University (approval number: 02094, approval date: 1 April 2020).

### 2.2. Establishment of Emphysema Model and FLCs Transfer

The mice were administered neutrophil elastase (NE) 10 U i.t. to generate the emphysema model. To investigate the distribution of transferred cells within the lungs of recipient mice, we used C57BL/6-Tg (CAG-EGFP) mice as FLC donors. The male and female donor mice were bred for 14 days. The bred mice were euthanized before delivery of offspring, and the lungs were harvested from the fetal mice. The fetal lung tissue was homogenized through a 70-μm mesh in ice-cold phosphate-buffered saline. The homogenate was washed and the cells were collected. In twenty-four hours (24 h) after the NE application, the FLCs were transferred 2 × 10^6^ cells/mouse i.t. into the emphysema model mice. FLCs are whole fetal lung cells that include several kinds of immature cells that are expected to differentiate into each structure of the lung.

### 2.3. Cellular Analysis in BALF

At 1 week and 4 weeks after NE intratracheal instillation, the mice were euthanized and bronchoalveolar lavage (BAL) was performed three times per mouse (0.35 mL/time) with normal saline. The BAL fluid (BALF) was centrifuged, and the cells were washed and re-suspended in the normal saline. The total number of cells was counted by hemocytometer with Turk solution (Wako Pure Chemical Industries, Ltd., Osaka, Japan). GFP-positive cells were observed by fluorescence microscopy.

### 2.4. Analysis of Lung Histopathology

After performing BAL, the lungs were removed from the chest and inflated with 500 μL of 10% formalin in PBS, followed by continuous inflation for 24 h at 10 cm H_2_O pressure. The lung tissues were dehydrated in ethanol, embedded in paraffin, sectioned (5 μm), and stained with hematoxylin–eosin staining. For the evaluation of emphysema, mean linear intercept (MLI) in three lung sections per mouse was measured as previously described [9]. MLI of the alveolar space was measured by drawing random test lines on sections of the lung and measuring the lengths separated by the alveolar septa; MLI is expressed as the mean length of the lines between sections. FLOVEL Filing System was used to evaluate the MLI (FLOVEL, Co., Ltd., Tokyo, Japan).

### 2.5. Investigation of Motility by FST

Forced swim test (FST) was performed at 4 weeks to investigate the motility function of the mice. The mice were allowed to swim in the aquarium for 6 min and the motility time was measured as previously described [10]. Motility time was defined as how long the mice moved their limbs. We measured the time by taking videos while the mice were swimming.

### 2.6. Statistical Analysis

Differences between the two groups with NE administration, i.e., transfer of fetal lung cells (FLCs) (+) and (−), were analyzed using an unpaired Student’s *t*-test. The significance of the differences among the three groups consisting of the two groups with NE administration and one without NE administration was determined by analysis of variance (ANOVA) with Bonferroni correction. All experiments were performed at least twice with 4 or more animals per group. Data are expressed as mean ± SEM, and *p* < 0.05 was considered to indicate significance.

## 3. Results and Discussion

**Figure 1:**
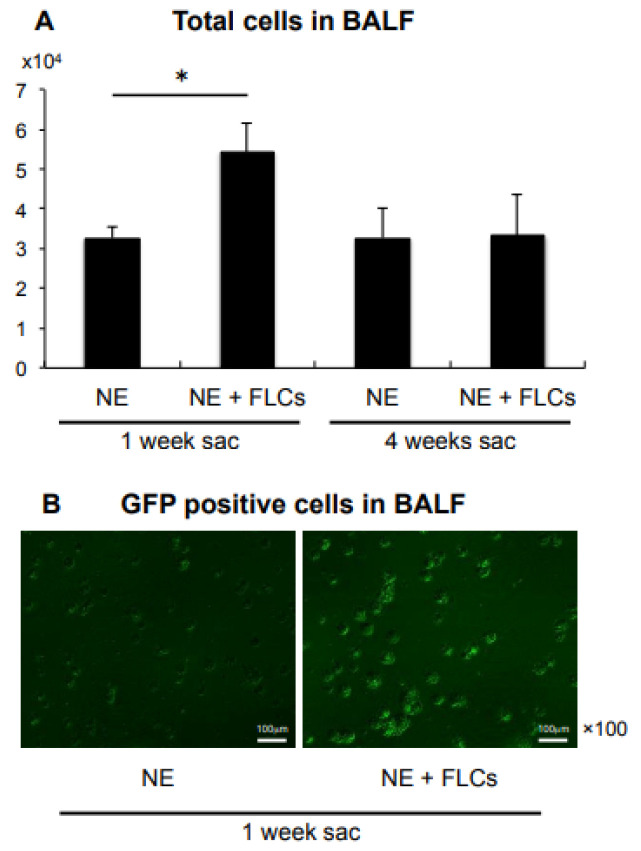
Analysis of total number of cells in the BALF at 1 week and 4 weeks after NE administration. (**A**) Representative slides of BALF total cells showing under a fluorescence microscope from 1 week after the NE administration with or without FLCs transfer. (**B**) * *p* < 0.05, *n* = 6–8. BALF: bronchoalveolar lavage fluid, NE: neutrophil elastase, FLCs: fetal lung cells, sac: sacrifice.

The number of total cells in the BALF was significantly increased in the FLCs-transferred group (*n* = 8) compared with the non-transferred group (*n* = 7) at 1 week after NE instillation (number of total cells: 5436 ± 419 vs. 2936 ± 231 (mean ± standard error of the mean); *p* < 0.001, *n* = 8 and 7, respectively, Figure 1A). In contrast, there was no significant difference in cell counts between the transferred and non-transferred groups at 4 weeks after NE instillation (number of total cells: 3471 ± 571 vs. 3017 ± 368, *p* = 0.518, *n* = 7 and 6, respectively, Figure 1A).

This result suggests that FLCs remained in the alveolar space at 1 week and had not yet engrafted into the alveolar wall or lung parenchyma. At 4 weeks, as the FLCs had likely engrafted into the alveolar wall or lung parenchyma, the number of total cells and monocytes observed in the BALF of the FLCs-transferred group decreased to the number comparable with the non-transferred group. Fluorescence microscopy revealed that a large number of GFP-positive BALF cells derived from the transferred FLCs were observed at 1 week (Figure 1B), whereas these cells were scarcely present at 4 weeks.

**Figure 2:**
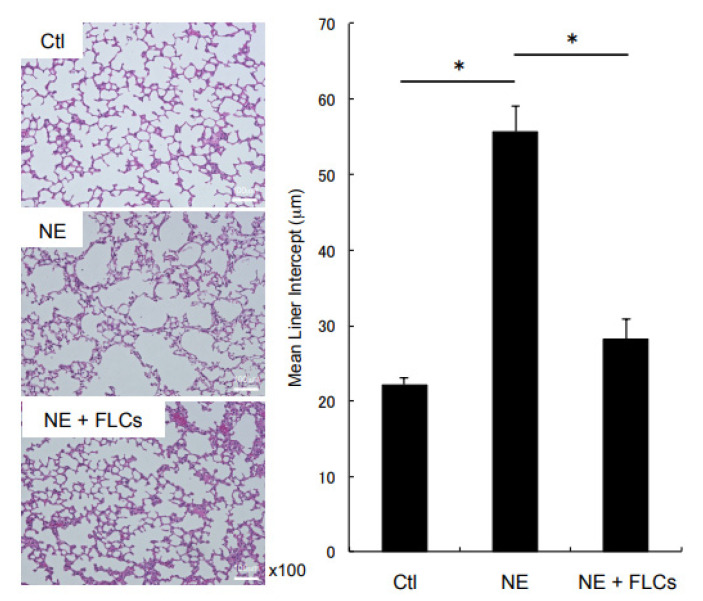
Representative lung sections showing H&E staining from control and 4 weeks after NE administration with or without FLCs transfer. The size of the alveolar space was evaluated by measurement of MLI. * *p* < 0.05, *n* = 8 for each. H&E: hematoxylin and eosin, Ctl: control, NE: neutrophil elastase, FLCs: fetal lung cells, MLI: mean liner intercept.

Histological evaluation of emphysema, using the measurement of MLI, revealed no significant difference among the three groups regardless of NE administration and FLCs-transfer at 1 week. However, at 4 weeks, the instillation of NE showed a significant increase in the MLI compared to the non-instillation group (Figure 2), which confirmed that the intratracheal instillation of NE caused the destruction of the alveolar walls and induced emphysematous changes as previously reported [4]. In comparison between the NE-administered groups, the MLI was significantly shorter in the FLCs-transferred group at 4 weeks (Figure 2). These findings might suggest that the transfer of FLCs inhibited the destruction of alveolar walls and the development of emphysema.

**Figure 3:**
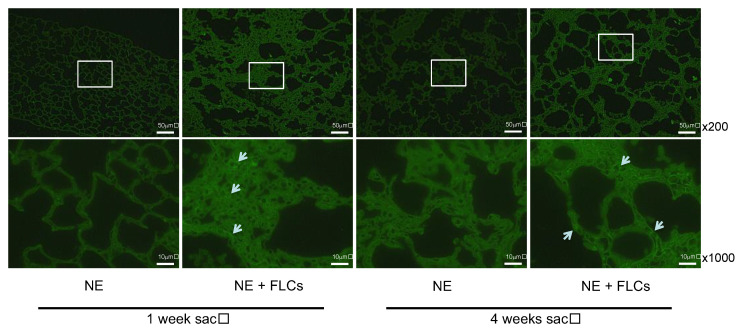
Representative lung sections showing under a fluorescence microscope from 1 week or 4 weeks after the NE administration with or without FLCs transfer. NE: neutrophil elastase, FLCs: fetal lung cells sac: sacrifice. White box shows the enlarged part and the arrows indicate GFP-positive cells.

Attempts were made to identify the origin of the cells that reconstructed the damaged alveolar structure by employing the green mouse as the donor of embryonic cells obtained from the lung tissue. We hypothesized that the cells having grown at the damaged alveoli could be derived from the donor embryonic cells, which would be recognized as green under a fluorescence microscope. However, we were not able to reveal if the cells were derived from the donor since the green was not bright enough to distinguish the cells of the donor of green mice from the cells of the recipient of non-green mice. Briefly, under fluorescence microscopy, the GFP-positive cells appeared seeming to have aggregated mainly in the lung parenchyma at 1 week and in the alveolar walls at 4 weeks (Figure 3). As mentioned above, some uncertainties were noted. One is due to an unclear distribution of transferred cells because of the weakness of fluorescence. For this reason, not only did the transferred cells themselves proliferate and repair the emphysema, but it is also possible that the host lung cells were stimulated by the transferred cells to repair the emphysema. Because FLCs were transferred 24 h after the elastase instillation, which is before the emphysematous change was established, the mechanisms of the improvement of emphysema might not only be caused by the direct contribution of FLCs but also by several indirect factors such as the inhibition of proteolysis or other anti-inflammatory effects. However, the possibility that the FLCs might be a source of anti-protease action is unlikely since alpha1-anti-trypsin (AAT), the major physiological proteinase inhibitor, is produced by liver hepatocytes in a steady-state manner [11]. For the characteristics of FLCs, Yamamoto et al. reported that fetal mouse lung mesenchymal cells are able to differentiate to vascular encothelia, osteogenic and chondrogenic cell lineages by various types of stimuli in vitro [12]. Moreover, Shibuya et al. reported that whole fetal lung culture expressed key genes such as E-cadherin and collagen-4 and established recapitulating branching morphogenesis. Additionally, the culture of mesenchyme-free epithelium rudiments, were able to generate new branches [13].

**Figure 4:**
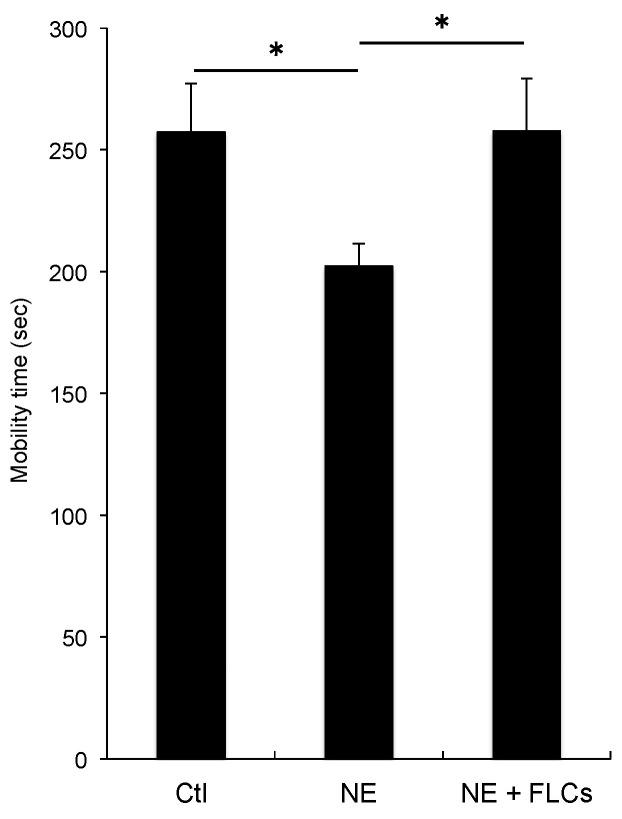
Analysis of motility time in 6 min at 4 weeks after the NE administration with or without FLCs transfer. * *p* < 0.05, *n* = 6 for each. NE: neutrophil elastase, FLCs: fetal lung cells.

The investigation of motility by FST showed a smaller amount of exercise in the NE-administered group compared with the non-administered group at 4 weeks (motility time in 6 min: 202.2 ± 9.32 s vs. 257.3 ± 19.9 s; *p* = 0.040; *n* = 6 for each, Figure 4). The transfer of FLCs significantly improved the amount of exercise in the NE-administered groups (motility time in 6 min: 257.8 ± 0.12 s vs. and 202.2 ± 9.32 s; *p* = 0.049; *n* = 6 for each, Figure 4). This result indicates that FLCs-transfer could increase exercise capacity, and further suggests that this approach might reduce the extent of emphysematous changes, as reflected in the relatively larger amount of the exercise.

As results, the fact we have confirmed is that the alveolar damage by elastase in our murine emphysema model has partially reconstituted by transferring embryonic lung cells, and the pulmonary function of oxygenation has been assessed to be improved by measuring the duration of exhaustion in water.

This study has some limitations. First, the study was based on an NE-induced model with young mice, which displays emphysematous changes without the accompanying chronic inflammation caused by cigarette smoke and/or immune aging. Future studies aiming to validate the present findings should involve other emphysema models. Second, FLCs do not constitute a single cell type, and the involvement of individual cell types remains unclear. It has been shown that lung progenitor cells were purified from mouse embryonic stem cells, which might be the next candidate cells for transfer [14,15]. Additionally, instead of transferring FLCs, future studies might utilize induced pluripotent stem (iPS) cells or other immature cells before their differentiation into specific cell types. Third, respiratory function was analyzed by FST in this study. However, it might be better to measure the diffusing capacity for carbon monoxide (DLCO), as previously reported [16], or measure other respiratory functions such as static compliance by using a plethysmograph chamber (e.g., Buxco Electronics Inc., Troy, NY, USA). This would be another important future study.

Finally, in the present study, we attempted to improve the emphysematous changes by transferring FLCs, but this method is difficult to translate directly into a clinical approach. To apply the results to clinical practice, it is important to investigate the molecular characterization of the FLCs and identify which are the key molecular factors involved in lung regeneration.

## 4. Conclusions

In conclusion, using a murine emphysema model, we have shown that the transfer of FLCs, presumed to be progenitor cells of the lung tissue, could improve emphysematous changes in the lung. This improvement was observed in histological specimens and exercise capacity. These preliminary findings suggest that COPD patients might benefit from the transferring of immature lung progenitor cells directly into the airway or alveoli, where the transferred cells might support the improvement of emphysema. Moreover, it is expected to recover the respiratory failure and increase activity.

## Data Availability

The data that support the findings of this study are available from the corresponding author upon reasonable request.
